# Food-related illness and death in the United States.

**DOI:** 10.3201/eid0506.990624

**Published:** 1999

**Authors:** C Hedberg


correctly identified *E. faecium* and *E. faecalis* to the species level, most (4 of 5) did not correctly identify *E. gallinarum* (three misidentified it as *E. casseliflavus* and one as *E. faecalis*).

The results of this study are consistent with those of previous studies in the United States (4,5), South America (6), Spain (7), and Mexico (8). Although in countries like Chile, disk diffusion is practical and reliable for most susceptibility testing, detecting low-level vancomycin resistance in enterocci is difficult without supplementary testing. In Chile, as in other countries, strategies should be implemented to improve detection of these strains, including improvement of phenotypical and genotypical methods for VRE detection and species identification. Documentation of proficiency in detecting VRE is important for improving laboratory performance, detecting clinical isolates, and accurate and reliable reporting to local, national, and international surveillance systems.

**Jaime A. Labarca,* L. Clifford McDonald,† María Eugenia Pinto,‡ Elizabeth Palavecino,*‡ Patricia González,‡ Erna Cona,‡ Alejandra Fernández,‡ María Soledad Giglio,‡ and William R. Jarvis†**

*P. Universidad Católica de Chile, Santiago, Chile; †Centers for Disease Control and Prevention, Atlanta, Georgia, USA; and ‡Chilean Society for Infectious Diseases, Santiago, Chile

### References

1. Marin ME, Mera JR, Arduino RC, Correa AP, Coque TM, Stambulian D, et al. First report of vancomycin-resistant *Enterococcus faecium* isolated in Argentina. Clin Infect Dis 1998;26:235-6.
2. Cereda RF, Medeiros EA, Vinagre A, Rego ST, Hashimoto A, Febre N, et al. Epidemiologic analysis for acquisition of vancomycin-resistant enterococcus (VRE) in an intensive care unit in Brazil. In: Proceedings of the Eighth Annual Meeting of the Society for Healthcare Epidemiology of America; 1998; São Paulo, Brazil.
3. National Committee for Clinical Laboratory Standards (NCCLS). Performance standards for antimicrobial disk susceptibility tests: approved standard M2-A6, 6th ed. Villanova (PA): The Committee.
4. Tenover FC, Tokars J, Swenson J, Paul S, Spitalny K, Jarvis WR. Ability of clinical laboratories to detect antimicrobial agent-resistant enterococci. J Clin Microbiol 1993;31:1695-9.
5. Rosenberg J, Tenover FC, Wong J, Jarvis W, Vugia DJ. Are clinical laboratories in California accurately reporting vancomycin-resistant enterococci? J Clin Microbiol 1997;35:2526-30.
6. Cookson ST, Lopardo H, Marin M, Arduino R, Rial MJ, Altschuler M, et al. Study to determine the ability of clinical laboratories to detect antimicrobial-resistant *Enterococcus* spp. in Buenos Aires, Argentina. Diagn Microbiol Infect Dis 1997;29:107-9.
7. Alonso-Echanove J, Robles B, Jarvis WR. Proficiency of clinical laboratories in Spain in detecting vancomycin-resistant *Enterococcus spp*. The Spanish VRE Study Group. J Clin Microbiol 1999,37:2148-52.
8. McDonald LC, Garza LR, Jarvis WR. Proficiency of clinical laboratories in and near Monterrey, Mexico, to detect vancomycin-resistant enterococci. Emerg Infect Dis 1999;5:143-6.

### Food-Related Illness and Death in the United States

**To the Editor:** Dr. Mead and colleagues should be commended for attempting to estimate the prevalence of foodborne disease in the United States (1). Their study provides more complete estimates than previous studies in terms of the number of foodborne pathogens included; for example, it includes the first realistic estimate of the number of cases of disease due to Norwalk-like caliciviruses. However, the publication of these estimates raises some important issues.

Even though "accurate estimates of disease burden are the foundation of sound public health policy" (2), most of these estimates (in particular, the assumption that unknown agents are transmitted by food in the same proportion as known agents) were derived from assumptions rather than data. Known foodborne agents clearly cannot account for most gastrointestinal illnesses (1). However, illnesses from unknown agents may be as likely to have the transmission characteristics of rotavirus (1% foodborne) or *Cryptosporidium* (10% foodborne) as those of the Norwalk-like viruses (40% foodborne). Furthermore, it was assumed that detecting outbreaks or cases of toxin-mediated illnesses (e.g., due to *Bacillus cereus*, *Staphylococcus aureus*, or *Clostridium perfringens)* follows the model of *Salmonella*. In the authors' entire list of known foodborne agents, data are presented for cases identified both from outbreaks and active surveillance for only three agents: *Salmonella, Shigella*, and *Campylobacter*. *Salmonella* is clearly the most highly characterized, hence the most attractive as a model. However, the ratios of the numbers of cases detected through active surveillance to the numbers of cases detected through outbreaks range from 10 for *Salmonella* to more than 400 for *Campylobacter*. What if the ratios for toxin-mediated illnesses were more similar to *Campylobacter* than to *Salmonella* ratios? The total estimated cases of these illnesses would increase by a factor of 40. The inadequacy of simply applying a *Salmonella*-based multiplier to the number of cases reported from outbreaks can be demonstrated by applying that multiplier to the total number of cases reported in all foodborne disease outbreaks, typically 15,000 to 20,000 per year (3,4). On the basis of these estimates, the number of foodborne illnesses would range from 5.7 million to 7.6 million, including illnesses caused by unknown agents.

The authors make similar assumptions for hospitalizations and deaths: unknown agents are estimated to account for 81% of hospitalizations and 65% of deaths due to foodborne illnesses. In a retrospective review of death certificate data similar to that used by Mead and colleagues, Perkins et al. projected the number of unexplained deaths possibly due to infectious diseases they expected to find in the Emerging Infections Program sites (5). Prospectively, a much smaller number of unexplained deaths was actually found, because known causes were identified through a detailed review of the death certificates and cases (6). A prospective examination of death certificates for foodborne diseases might also result in a smaller than expected yield.

The need to rely on assumptions to generate estimates highlights the gaps in our understanding of foodborne diseases. A dozen different studies could address these data gaps. However, once the 76 million figure is agreed upon, the perceived need for these studies will decrease.

Finally, if these estimates are accepted as reasonable, do current food safety efforts represent sound public policy? If 82% of foodborne illnesses, 81% of hospitalizations, and 65% of deaths are caused by agents we have not yet identified, where is the commitment of resources needed to identify them? If eradicating *Campylobacter*, *Salmonella, Escherichia coli* O157:H7, and *Listeria* would reduce the number of foodborne illnesses by only 5%, hospitalizations by 10%, and deaths by 25%, why are these agents the primary focus of our national foodborne disease control efforts? Overestimating the occurrence of foodborne diseases caused by unknown agents may lead us to undervalue the public health importance of these and other well-known agents.

Estimating the occurrence of foodborne diseases is daunting. The numerous efforts, including this one by Mead et al., to provide estimates have serious shortcomings. The real challenge is to identify the gaps in our knowledge so that they can be systematically addressed and updated estimates of foodborne illness can be provided to guide prevention efforts and assess the effectiveness of current food safety measures (2).

**Craig Hedberg**
University of Minnesota,
Minneapolis, Minnesota, USA

### References

1. Mead PS, Slutsker L, Dietz V, McCaig LF, Bresee JS, Shapiro C, et al. Food-related illness and death in the United States. Emerg Infect Dis 1999;5:607-25.
2. Centers for Disease Control and Prevention. CDC data provides the most complete estimate on foodborne disease in the United States. Press release available at URL: http://www.cdc.gov/od/oc/media/pressrel/r990917.htm
3. Foodborne disease outbreaks, 5-year summary, 1983-1987. MMWR Morb Mortal Wkly Rep 1992;39(SS-1):1-15.
4. Surveillance for foodborne disease outbreaks. United States, 1988-92. MMWR Morb Mortal Wkly Rep 1996;45(SS-5):2-55.
5. Perkins BA, Flood JM, Danila R, Holman RC, Reingold AL, Klug LA, et al. Unexplained deaths due to possibly infectious causes in the United States: defining the problem and designing surveillance and laboratory approaches. Emerg Infect Dis 1996;2:47-53.
6. Minnesota Department of Health. Annual summary of communicable diseases reported to the Minnesota Department of Health, 1998. Disease Control Newsletter 1999;27:29-30.

### Food-Related Illness and Death in the United States–Reply to Dr. Hedberg

**To the Editor:** Like all scientific undertakings, our estimates require assumptions. Because the actual frequency of foodborne transmission of unknown agents cannot be measured directly, it must be assumed. If unknown agents had transmission characteristics similar to those of rotavirus (1% foodborne transmission) or cryptosporidium (10% foodborne transmission), as Dr. Hedberg suggests, the number of cases of foodborne illness caused by unknown agents would be substantially lower than we estimated. However, unknown agents could just as easily have the transmission characteristics of *Escherichia coli* O157:H7 or *Campylobacter* (80% foodborne transmission), which just 30 years ago

